# A comparative analysis of deep learning and hybrid iterative reconstruction algorithms with contrast-enhancement-boost post-processing on the image quality of indirect computed tomography venography of the lower extremities

**DOI:** 10.1186/s12880-024-01342-0

**Published:** 2024-07-01

**Authors:** Huayang Du, Xin Sui, Ruijie Zhao, Jiaru Wang, Ying Ming, Sirong Piao, Jinhua Wang, Zhuangfei Ma, Yun Wang, Lan Song, Wei Song

**Affiliations:** 1 Department of Radiology, Peking Union Medical College Hospital (PUMCH), Chinese Academy of Medical Sciences & Peking Union Medical College (CAMS & PUMC), No.1 Shuaifuyuan Wangfujing Dongcheng District, Beijing, 100730 China; 2 Canon Medical Systems (China), No.3, Xinyuan South Road, Chaoyang District, Beijing, 100027 China

**Keywords:** CT venography, Lower extremity, Deep vein thrombosis, Contrast enhancement boost

## Abstract

**Purpose:**

To examine whether there is a significant difference in image quality between the deep learning reconstruction (DLR [AiCE, Advanced Intelligent Clear-IQ Engine]) and hybrid iterative reconstruction (HIR [AIDR 3D, adaptive iterative dose reduction three dimensional]) algorithms on the conventional enhanced and CE-boost (contrast-enhancement-boost) images of indirect computed tomography venography (CTV) of lower extremities.

**Materials and methods:**

In this retrospective study, seventy patients who underwent CTV from June 2021 to October 2022 to assess deep vein thrombosis and varicose veins were included. Unenhanced and enhanced images were reconstructed for AIDR 3D and AiCE, AIDR 3D-boost and AiCE-boost images were obtained using subtraction software. Objective and subjective image qualities were assessed, and radiation doses were recorded.

**Results:**

The CT values of the inferior vena cava (IVC), femoral vein ( FV), and popliteal vein (PV) in the CE-boost images were approximately 1.3 (1.31–1.36) times higher than in those of the enhanced images. There were no significant differences in mean CT values of IVC, FV, and PV between AIDR 3D and AiCE, AIDR 3D-boost and AiCE-boost images. Noise in AiCE, AiCE-boost images was significantly lower than in AIDR 3D and AIDR 3D-boost images ( *P* < 0.05). The SNR (signal-to-noise ratio), CNR (contrast-to-noise ratio), and subjective scores of AiCE-boost images were the highest among 4 groups, surpassing AiCE, AIDR 3D, and AIDR 3D-boost images (all *P* < 0.05).

**Conclusion:**

In indirect CTV of the lower extremities images, DLR with the CE-boost technique could decrease the image noise and improve the CT values, SNR, CNR, and subjective image scores. AiCE-boost images received the highest subjective image quality score and were more readily accepted by radiologists.

## Introduction

The incidence of deep vein thrombosis (DVT) has significantly increased over the last two decades, affecting 20–36% of DVT patients. This occurrence is commonly associated with recurrent venous thromboembolism and pulmonary embolism [1]. Beyond ultrasonography, indirect computed tomography venography (CTV) of the lower extremities offers accurate diagnosis and evaluation of DVT. Adequate enhancement of veins on CTV images is crucial for accurately identifying deep vein thrombosis. Unlike CT angiography, CTV often exhibits suboptimal vascular enhancement, primarily attributed to the passage of the contrast agent through the systemic circulation. To reduce radiation exposure and improve CT enhancement, lower tube voltage protocols such as 80 kVp or 100 kVp, in combination with iterative reconstruction, are frequently utilized in lower extremity CTV, as well as body and chest CT scans [2–5]. However, the venous enhancement effect is not suitable due to various factors such as patient weight, limb swelling, and venous reflux, and valve insufficiency. The CT values of venous blood and clots are slightly different [6], affecting the radiologists’ diagnostic confidence.

Despite the combined use of an iterative algorithm, low tube voltage, and high contrast concentration, some patients still encounter inadequate venous enhancement, thereby hindering the fulfillment of the diagnostic criteria. Re-examination exacerbates the situation by exposing patients to higher levels of radiation. Furthermore, re-injection of contrast medium (CM) can trigger a response to potential contrast-induced nephropathy (CIN).

Contrast-enhancement-boost (CE-boost) is an innovative post-processing technique that improves vascular enhancement by increasing the intensity of the vessels retrospectively without altering the amount of contrast medium (CM) or the CT scanning protocol [7]. This approach relies on precise deformable registration algorithms developed explicitly for non-contrast and contrast-enhanced CT images. The CE-boost process involves several steps: First, an iodine image is generated by subtracting a non-contrast CT image from a contrast-enhanced image. Subsequently, a noise-reduced iodine image is obtained through a noise-reduction procedure applied to the subtracted iodine image. Finally, a CE-enhanced image is created by adding the noise-reduced image to the original contrast-enhanced image using an automated process of pixel alignment [8–10].

AiCE (Advanced Intelligent Clear-IQ Engine, AiCE), an advanced deep learning reconstruction (DLR) algorithm developed by Canon Medical Systems, effectively addressed the limitations and shortcomings associated with iterative reconstruction techniques, such as image smoothing and the dependency of spatial resolution on contrast and dose level [1]. Several studies have supported significant dose reductions, up to 80%, and reduced image noise in various anatomical regions, including the chest, abdomen, and coronary CT angiography, with the implementation of low-dose CT with AiCE [11–13]. To our knowledge, no studies have been reported on using AiCE for indirect CTV in the lower extremities.

This research aims to examine whether there is a significant difference in image qualities of indirect CTV of the lower extremities at 80 kVp with a CM volume (90 mL) of iodine concentration CM (370 mg/mL) between the hybrid iterative reconstruction(HIR) AIDR 3D (adaptive iterative dose reduction three dimensional), and deep learning reconstruction (DLR) AiCE reconstruction algorithms on the conventional enhanced and those using CE-boost technique (AiCE-boost and AIDR 3D-boost).

## Materials and methods

### Patient selection

This retrospective study included 70 consecutive patients (42 women and 28 men; mean age, 49.33 ± 15.50 years; range, 29-78 years; mean body mass index (BMI), 24.64 ± 3.67 kg/m2). These patients underwent clinically indicated contrast-enhanced CTV imaging at Peking Union Medical College Hospital between June 2021 and October 2022. The clinical indications for CTV in patients were suspected deep vein thrombosis, varicose veins, and lower extremity swelling. The exclusion criteria for patients were as follows: patients with severe hepatic or renal insufficiency, cardiac insufficiency, contrast nephropathy, iodine contrast allergy, history of internal or external fixation or amputation, and pregnant women. The study and protocol underwent thorough evaluation and obtained approval from the ethical committee at our hospital.

### CT protocols and image reconstruction

#### CT protocols

All study participants were scanned with a 320-row detector CT scanner (Aquilion ONE GENESIS Edition; Canon Medical Systems). The acquisition of non-enhanced without intravenous iodine contrast and enhanced images with intravenous iodine contrast adhered to a standardized scanning protocol recommended by the CT scanner manufacturer. Scanning was performed in the craniocaudal direction from the T8 level to the ankles, with participants instructed to maintain a breath-hold in the inspiratory phase. Other acquisition parameters were as follows: 80 kVp; automatic tube current modulation (ATCM) [XY-Modulation, 150-440 mA]; fixed standard deviation (SD) = 8.8 for image thickness 5.0 mm; pitch factor 0.813, helical pitch 65.0; collimation 0.5 mm×80. The non-enhanced images were acquired first. Then, enhanced images were acquired. After the administration of 90 mL of nonionic, iso-osmolar iodinated contrast medium (Ultravist 370, 370 mg/mL, Bayer Pharma AG, Shanghai, China) via intravenous injection at a rate of 3 mL/s through an antecubital vein, followed by 30 mL of 0.9% saline solution at a flow rate of 3 mL/s, contrast-enhanced CT images were acquired. The CTV scan was performed approximately 180 s after the administration of intravenous CM [2, 14].

#### Image reconstruction

The reconstructed images, including non-enhanced and enhanced images, were categorized into four groups. Group A was reconstructed using AIDR 3D (FC08), whereas Group B was reconstructed using AiCE (body sharp kernel) [15]. All reconstructions were performed with a slice thickness of 1.0 mm and a slice interval of 0.8 mm, with a pixel matrix of 512 × 512. Subsequently, Canon post-processing software (^sure^Subtraction Iodine Mapping, Canon Medical Systems) was utilized for subtraction processing. The Group C (AIDR 3D-boost) images were generated by subtracting the AIDR 3D non-enhanced images from the AIDR 3D enhanced images. AiCE images underwent a similar processing procedure to generate the Group D (AiCE-boost) images. Four groups of images were sent to the Cannon workstation (Vital, version 4.0.693) for objective assessment. The picture archiving and communication system (PACS) was used for subjective evaluations.

### Image analysis

#### Objective assessment of image quality

Objective indicators were measured by a radiologist using the Vital workstation (version 4.0.693). The radiologist measured the mean CT values (Hounsfield units, HU) and SD of CT attention of each vein, and SD served as an indicator of image noise. The measurements were acquired at precise anatomical locations, comprising the inferior vena cava (IVC, area of 100–150 mm^2^) at the level of the L4 vertebra, the right femoral vein (RFV, area of 20–50 mm^2^) at the level of the greater trochanter of the femur, and the right popliteal vein (RPV, area of 20–50 mm^2^) at the level of the popliteal fossa. Region of interest (ROI) was manually sketched on one group of images and copied ROIs to the other three groups to ensure they were the same size and location. The circular ROI was positioned at the central area of the lumen, excluding vessel walls from the ROI. An ROI (size 80–110 mm^2^) was placed in the medial adductor muscle of the right mid-thigh. Three measurements were taken at each position, and the mean value was calculated. The signal-to-noise ratio (SNR) and contrast-to-noise ratio (CNR) of each vein were computed using the following formulas [16]:

SNR = CT_lumen_/ SD_lumen_.

CNR = CT_lumen_ -CT_muscle_/ SD_lumen_.

#### Subjective assessment of image quality

Two radiologists, one with 6 years (Reader A) and another with 11 years (Reader B) of work experience in CTV, were both double-blinded on image parameters, reconstruction methods, CT values, and noise. They assessed subjective image qualities using a 3-point scale for image noise based on literature references [3, 14], using scales of 5 and 5 points for venous enhancement, and diagnostic confidence in DVT [14, 17]. Overall image quality was evaluated by a 5-point scale, considering the image noise, venous enhancement, and diagnostic confidence in DVT [2, 3, 14, 18] (Table 1 for detailed criteria). Image analysis used transverse images with a window level (WL) of 40 HU and a window width (WW) of 400 HU. The readers were allowed to adjust W/L as in a real clinical scenario. The arrangement of the images on the PACS was 2 × 2, with the sequence of images randomly distributed, the patient information hidden, the reconstruction of the image, and whether it was a CE-boost image.

**Table 1:** Scores used for the subjective scoring of image quality characteristics

Scores	Description
Image noise	
1	Unacceptable, no diagnosis possible
2	Moderate, but sufficient for diagnosis
3	Optimal, none perceivable
Venous enhancement	
1	Less than adjacent muscular enhancement
2	Similar to adjacent muscular enhancement
3	Greater muscular enhancement but less than adjacent arterial enhancement
4	Similar to the adjacent arterial enhancement
5	Intravenous reinforcement better than 4
Diagnostic confidence in DVT	
1	Very poor
2	Poor
3	Average
4	High
5	Excellent
Overall image quality	
1	Unacceptable, no diagnosis possible
2	Poor, inadequate for diagnosis of the presence or absence of a clot
3	Fair, enhancement sufficient for diagnosis
4	Good, optimal enhancement allowing confident diagnosis of the presence or absence of a clot
5	Excellent, optimal enhancement superior to a score of 4 allowing for confident diagnosis of the presence or absence of a clot

The assessment procedure involved evaluating image noise, followed by venous enhancement, confidence in DVT, and overall image quality for all patients. Two radiologists evaluated the subjective outcomes independently, completing the evaluation of all indications within a week. After a one-month washout period, the aforementioned parameters were reassessed.

#### Detection of DVT in CTV of the lower extremities

Thrombi were defined as areas of reduced density within a blood vessel, either partially or entirely obstructing the lumen, and were surrounded by a dense ring of enhanced blood that was seen on two or more consecutive transverse images [19]. The veins assessed include inferior vena cava, common iliac vein, external iliac vein, internal iliac vein, common femoral vein, deep femoral vein, superficial femoral vein, popliteal vein, anterior tibial vein, posterior tibial vein, fibular vein. The presence or absence of thrombus was diagnosed on the four groups of images using a consultative method by the two radiologists mentioned above, recording the exact location of the vein containing the thrombus on a structured data form. The results of the diagnosis made by the two radiologists were used as a reference standard. Following a one-month washout, two radiologists evaluated the thrombi after reviewing each series of images independently.

### Evaluation of radiation dose

The CT dose index volume (CTDIvol) and dose-length products (DLPs) from the scanner system were recorded and documented in the dosage report.

### Statistical analysis

Statistical analyses were conducted using SPSS ver. 22.0.0 (SPSS Inc., Chicago, IL, USA). Continuous numerical variables were expressed as mean ± standard deviation if they conformed to normal distribution after Kolmogorov-Smimov test, or data were expressed as [median (Quartile1, Quartile4)] if they do not conform to normal distribution. One-way ANOVA was used to test the continuous variables data from the 4 groups of images if the data met variance chi-square, followed by Bonferroni post-hoc corrections. The Wilcoxon rank-sum test was used to test variable data with heterogeneous variance. Ordinal variables (qualitative grading) assessed by radiologists A and B were tested for normality using the Shapiro-Wilk test. For normally distributed data, the significance of intergroup differences was determined using the two-tailed Student’s t test. The non-normally distributed data were expressed as the median (minimum, maximum), and Friedeman was used to compare ordinal variables among the four groups’ images. *P* < 0.05 was considered statistically significant. The Cohen’s kappa coefficients (CKCs) were used to assess the consistency of subjective assessments between two observers and before and after for the same interobserver (Reader A, Reader B), respectively. CKCs values falling within the range of 0.00–0.20, 0.21–0.40, 0.41–0.60, 0.61–0.80, and 0.81–1.00 were categorized as slight, fair, moderate, substantial, and almost perfect agreement, respectively [20].

## Results

### Quantitative image quality

#### CT values

The mean CT values of IVC, FV, and PV in group A did not exhibit statistically significant differences compared to group B (all *P* > 0.05). Similarly, in group C, the mean CT values of IVC, FV, and PV showed no statistically significant differences from group D (all *P* > 0.05) (Table 2).

**Table 2:** CT attenuation and image noise of the AIDR 3D, AiCE, AIDR 3D-boost, and AiCE-boost images

			CT attenuation					Image noise	
	IVC		FV	PV		IVC		FV	PV
group A	146.65 ± 17.44		143.12 ± 18.84	126.01 ± 20.87		21.28 ± 3.74		19.90 ± 5.03	14.78 ± 3.92
group B	147.43 ± 17.60		144.05 ± 19.60	129.33 ± 20.96		12.47 ± 2.67		11.92 ± 2.95	9.54 ± 2.40
group C	199.01 ± 26.22		189.24 ± 27.20	166.18 ± 30.22		21.71 ± 5.65		20.03 ± 7.15	12.48 ± 5.43
group D	195.94 ± 35.46		189.81 ± 27.87	169.71 ± 30.72		8.99 ± 3.08		8.76 ± 3.38	6.88 ± 3.39
*P*-value	<0.001 ^a^		<0.001 ^a^	<0.001 ^a^		<0.001 ^a^		<0.001 ^a^	<0.001 ^a^
	P-value pairwise								
CT attenuation (IVC / FV / PV)									
	group A	group B			group C		group D		
group A	/	NS / NS / NS			<0.001 / <0.001 / <0.001		<0.001 / <0.001 / <0.001		
group B		/			<0.001 / <0.001 / <0.001		<0.001 / <0.001 / <0.001		
group C					/		NS / NS / NS		
group D							/		
Image noise (IVC / FV / PV)									
group A	/	<0.001 / <0.001/ 0.001			<0.001 / NS / 0.001		<0.001 / <0.001/ <0.001		
group B		/			<0.001 / <0.001/ <0.001		<0.001 / <0.001 / <0.001		
group C					/		<0.001 / <0.001 / <0.001		
group D							/		

Note. group A = AIDR 3D, group B = AiCE, group C = AIDR 3D-boost, group D = AiCE-boost. IVC = inferior vena cava, FV = femoral vein, PV = popliteal vein. NS = no significant differences. a The inter-group comparisons with significant differences (*P* < 0.05) were listed above

For group C, IVC, FV, and PV demonstrated an improvement in CT values of 52.36 ± 9.49 HU, 52.36 ± 9.49 HU, and 40.17 ± 10.30 HU, respectively. Group D images exhibited similarities to the group C images with no statistically significant differences in the improvements observed in the IVC, FV, and PV (*P* > 0.05). The CT values in IVC, FV, and PV indicated an estimated increase of 1.30 (range, 1.31–1.36) times in the CE-boost images.

#### Image noise

The mean image noise in group B was significantly lower than that of group A (*P* < 0.05). Group D images demonstrated the lowest noise level, with a statistically significant reduction in image noise compared to the group B (*P* < 0.05). However, there was no significant difference in image noise between group A and C (*P* > 0.05) (Table 2).

#### SNR and CNR

The mean SNR and CNR of group D images were the highest among the 4 groups in anatomical locations of IVC, FV, and PV; group A revealed the lowest SNR, CNR (all *P* < 0.05). In IVC and PV, group C displayed higher SNR and CNR compared to group B. for SNR and CNR, group B outperformed than group C in the anatomical location of FV. Nevertheless, no statistically significant differences existed between group B and C (*P*>0.05) (Table 3).

**Table 3:** Signal-to-noise ratio (SNR) and contrast-to-noise ratio (CNR) of the AIDR 3D, AiCE, AIDR 3D-boost, and AiCE-boost images

	IVC	FV	PV
(A) Signal-to-noise Ratio (SNR) of the AIDR 3D, AiCE, AIDR 3D-boost, and AiCE-boost images			
group A	7.14 ± 1.72	7.67 ± 2.29	4.01 ± 2.21
group B	9.98 ± 3.69	12.88 ± 5.07	8.81 ± 6.99
group C	12.26 ± 2.71	11.10 ± 5.86	14.50 ± 4.79
group D	24.83 ± 10.15	25.50 ± 12.10	32.15 ± 21.55
P-value	<0.001	<0.001	<0.001
(B) Contrast-to-noise ratio (CNR) of the AIDR 3D, AiCE, AIDR 3D-boost, and AiCE-boost images			
group A	3.64 ± 1.12	3.80 ± 1.39	4.01 ± 2.21
group B	5.98 ± 2.32	8.05 ± 16.28	6.37 ± 3.06
group C	6.22 ± 1.74	6.43 ± 3.54	8.81 ± 6.99
group D	14.71 ± 6.31	14.62 ± 7.48	17.08 ± 13.30
P-value	<0.001	<0.001	<0.001

Note. group A = AIDR 3D, group B = AiCE, group C = AIDR 3D-boost, group D = AiCE-boost.

IVC = inferior vena cava, FV = femoral vein, PV = popliteal vein

### Qualitative image quality

For subjective image noise, group D scored higher than group A and C, and group B obtained higher scores than group A (all *P* < 0.05). However, scores were not statistically different between groups A and C, B and D.

CE-boost demonstrated superior performance for the enhancing effect compared to the conventional enhanced images. No statistical differences were found between groups A and B, as well as between groups C and D.

In terms of image quality, group D showed the highest score, surpassing both group A and B, as well as group C (all *P* < 0.05). The mean score of group C was significantly higher than that of group B (Table 4).

**Fig. 1:**
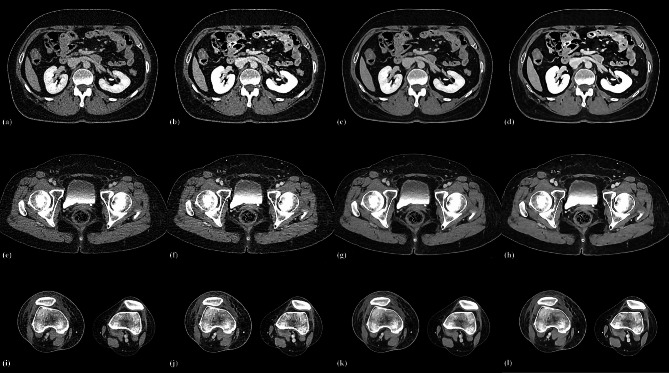
AIDR 3D, AiCE, AIDR 3D-boost and AiCE-boost axial images of a 52-year-old female in anatomical locations of the inferior vena cava (IVC), femoral vein (FV), and popliteal vein (PV). IVC (**a**-**d**), FV(**c**-**d**), PV(**i**-**l**). AIDR 3D (**a, e, i**), AiCE (**b**, **f**, **j**), AIDR 3D-boost (**c, g, k**) and AiCE-boost (**d, h, l**). The CE-boost images have increased vascular enhancement compared to the previous images. AiCE and AiCE CE-boost enhanced images provide more apparent vessel margins than AIDR 3D and AIDR 3D-boost images

**Fig. 2:**
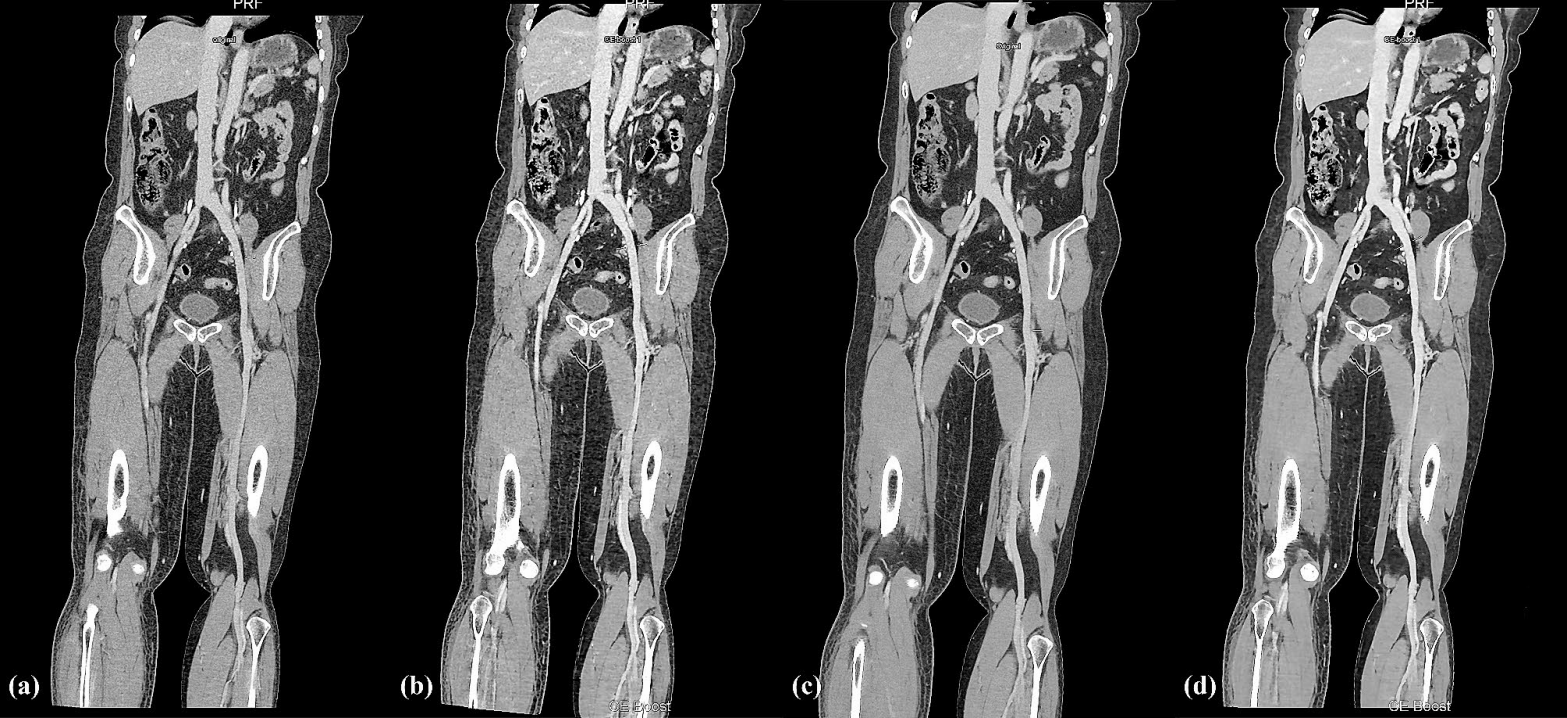
For the same patient, curved projection reformation (CPR) images inferior vena cava to the left popliteal vein. AIDR 3D (**a**), AiCE (**b**), AIDR 3D-boost (**c**) and AiCE-boost (**d**). The CPR images showed the entire length of the vein, and the vein margins and tissue AiCE and AiCE CE-boost enhanced images were more evident than AIDR 3D and AIDR 3D-boost images

**Fig. 3:**
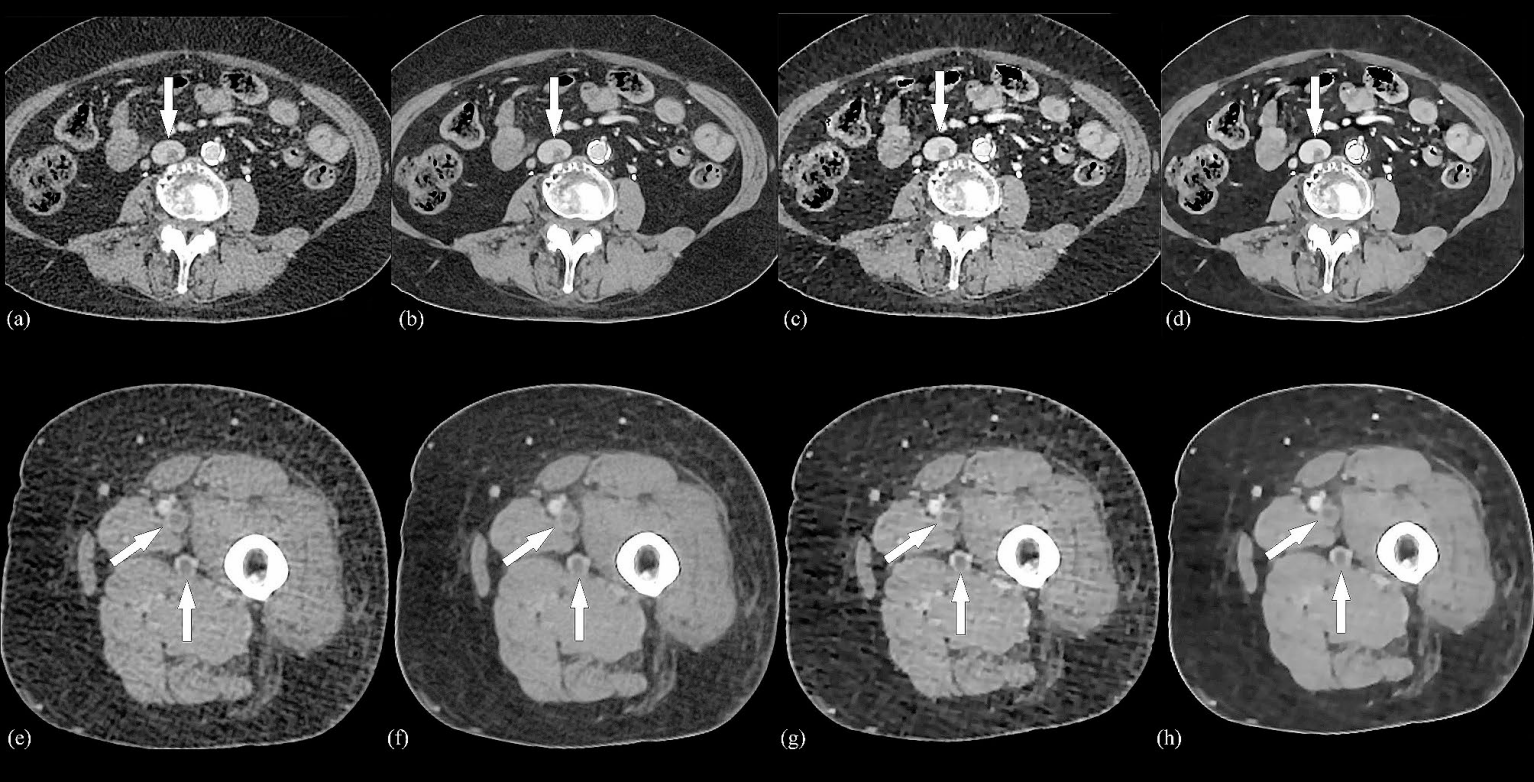
Axial CTV images of a 74-year-old patient with thrombosis in the inferior vena cava veins of the left lower extremity. AIDR 3D (**a**, **e**), AiCE (**b**, **f**), AIDR 3D-boost (**c**, **g**) and AiCE-boost (**d**, **h**). The clots within IVC (arrow, a-d), superficial femoral vein, and deep femoral vein in the upper left thigh(arrow, e-h)were well delineated and had a higher CT enhancement, SNR and CNR in AIDR 3D-boost, and AiCE-boost images than AIDR 3D and AiCE images. AiCE and AiCE-boost images showed a lower noise than AIDR 3D and AIRD 3D-boost images

For confidence in diagnosing thrombi, the group C and D scores were higher than those of group A and B, with the group D scores being the highest. The scores obtained from the AiCE assessment were found to be superior to those acquired in AIDR 3D (all *P* < 0.05). Three cases are displayed in Figs. 1 and 2, and 3.

### Thrombus distribution and radiologists’ diagnostic results

35 (35/70) patients were identified with the presence of a thrombus in their veins by two radiologists on 4 groups of images in a negotiated method. The anatomical distribution of the observed cases was as follows: inferior vena cava (*n* = 4), common iliac vein (*n* = 11), external iliac vein (*n* = 12), internal iliac vein (*n* = 10), common femoral vein (*n* = 14), superficial femoral vein (*n* = 24), deep femoral vein (*n* = 10), popliteal vein (*n* = 5), anterior tibial vein (*n* = 8), posterior tibial vein (*n* = 8), and fibular vein (*n* = 4).

The number of thrombi detected by radiologists A and B in the images of groups A, B, C and D was consistent with the two radiologists consultative results on inferior vena cava, common iliac vein, internal iliac vein, common femoral vein and popliteal vein.

In group A, radiologist A misdiagnosed 1 external iliac vein and 2 anterior tibial vein thrombus, and radiologist B misdiagnosed 1 external iliac vein and 1 anterior tibial vein thrombus. In group C, radiologist A misdiagnosed 1 anterior tibial vein and radiologist B misdiagnosed 1 anterior tibial vein thrombus.

**Fig. 4:**
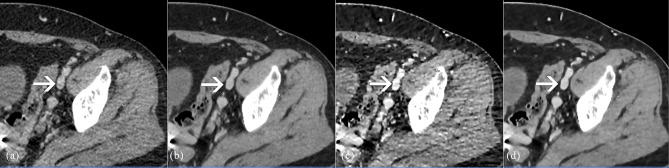
Axial CTV images of a 57-year-old male patient. AIDR 3D (**a**), AiCE (**b**), AIDR 3D-boost (**c**) and AiCE-boost (**d**). Suspicious filling defect in the left external iliac vein on the AIDR 3D image (arrow, a), and misdiagnosis of venous thrombosis by radiologists A and B, AiCE, AIDR 3D-boost, and AiCE-boost images clearly show no thrombosis in the lumen

**Fig. 5:**
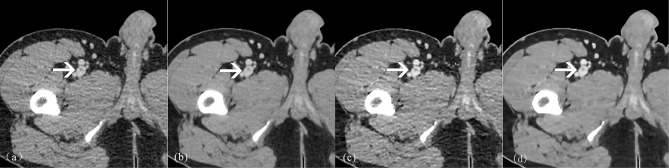
Axial CTV images of a 62-year-old male patient. AIDR 3D (**a**), AiCE (**b**), AIDR 3D-boost (**c**) and AiCE-boost (**d**). There was a suspicious filling defect in the right superficial femoral vein on the images of the AIDR 3D (arrow, **a**), and AiCE (arrow, **b**), which radiologists A and B missed on the AIDR 3D and AiCE image. The luminal filling defect is clearly shown on the AIDR 3D-boost image (arrows, **c**), and the AiCE-boost image (arrows, **d**) and was accurately diagnosed by radiologists A and B

In group A, radiologist A missed 6 superficial femoral vein, 2 deep femoral vein, and 1 fibular vein thrombus, and radiologist B missed 5 superficial femoral vein, 1 deep femoral vein, and 1 fibular vein thrombus. In group B, radiologist A missed 4 superficial femoral vein and radiologist B missed 4 superficial femoral vein thrombus. In group C, radiologist A missed 4 superficial femoral vein, 1 deep femoral vein, and 1 fibular vein thrombus, and radiologist B missed 4 superficial femoral vein and 1 fibular vein thrombus. In group D, radiologist A missed 4 superficial femoral vein and radiologist B missed 4 superficial femoral vein thrombus. Two cases are displayed in Fig. 4, and 5.

### Inter-reader and intra-reader agreement

The inter-reader agreement in evaluating subjective quality was deemed approaching perfection, as indicated by a kappa value ranging from 0.840 to 0.981 (Table 4).

**Table 4:** Subjective image analysis of the AIDR 3D, AiCE, AIDR 3D-boost, and AiCE-boost images

	Image noise (*n* = 70)		Venous enhancement (*n* = 70)		Image quality (*n* = 70)		Confidence of DVT (*n* = 70)	
	Score (Reader A/ Reader B)	Kappa (95% CI)	Score (Reader A/ Reader B)	Kappa (95% CI)	Score (Reader A/ Reader B)	Kappa	Score (Reader A/ Reader B)	Kappa
group A	2 (2,3) / 2 (2,3)	0.913 (0.817,1.000)	3 (2,4) / 3 (2,4)	0.959 (0.881,1.000)	3 (1,4) / 3 (1,4)	0.944(0.868,1.000)	3 (2,4) / 3 (2,4)	0.856(0.763,1.000)
group B	3 (2,3) / 2 (2,3)	0.881(0.651,1.000)	3 (2,4) / 3 (2,5)	0.908 (0.783,1.000)	4 (2,5) / 4 (1,5)	0.964(0.872,1.000)	4 (3,5) / 4 (3,5)	0.884(0.731,1.000)
group C	2 (2,3) / 2 (2,3)	0.966(0.899,1.000)	4 (2,5) / 4 (2,5)	0.975 (0.926,1.000)	4 (1,5) / 4 (1,5)	0.840(0.756,0.924)	4 (3,5) /5 (3,4)	0.845(0.639,1.000)
group D	3 (2,3) / 2 (2,3)	0.971(0.797,0.989)	4 (2,5) / 4 (2,5)	0.923 (0.839,0.998)	4 (3,5) / 4 (3,5)	0.869(0.759,0.976)	5 (4,5) /5 (4,4)	0.842(0.708,0.920)
P, value overall^★^	<0.001 / <0.001		<0.001 / <0.001		<0.001 /<0.001		<0.001 / <0.001	
P, value pairwise^◇^								
	**Image noise (Reader A / Reader B)**				**Venous enhancement (Reader A / Reader B)**			
	**group A**	**group B**	**group C**	**group D**	**group A**	**group B**	**group C**	**group D**
group A	/	<0.001 / <0.001	0.142 / 0.147	<0.001 / <0.001	/	1.000 / 1.000	<0.001 / <0.001	<0.001 / <0.001
group B		/	<0.001 / <0.001	1.000 / 1.000		/	<0.001 / <0.001	<0.001 / <0.001
group C			/	<0.001 / <0.001			/	1.000 / 1.000
group D				/				/
	**Image quality (Reader A/ Reader B)**				**Confidence of DVT (Reader A/ Reader B)**			
	group A	**group B**	**group C**	**group D**	**group A**	**group B**	**group C**	**group D**
group A	/	<0.001 / <0.001	<0.001 / <0.001	<0.001 / <0.001	/	<0.001 / <0.001	<0.001 / <0.001	<0.001 / <0.001
group B		/	<0.001 / <0.001	<0.001 / <0.001		/	<0.015 / <0.013	<0.001 / <0.001
group C			/	<0.001 / <0.001			/	<0.001 / <0.001
group D				/				/

Note. group A = AIDR 3D, group B = AiCE, group C = AIDR 3D, boost, group D = AiCE, boost. ^★^ The inter, group comparisons with significant differences (*P* < 0.05) were listed above. ^◇^ Friedman test for pairwise comparison of *P*,values between groups

The intra-reader agreement [kappa (95% CI)]of radiologist A in evaluating the image noise, venous enhancement, image quality, and confidence of DVT was almost perfect [0.885 (0.746, 0.966), 0.943 (0.923, 0.962), and 0.920 (0.842, 0.987), respectively, in Group A; 0.870 (0.837, 0.903), 0.921 (0.829, 1.000), and 0.913 (0.829, 0.997) in Group B; 0.923 (0.890, 0.956), 0.911 (0.833, 0.989), and 0.893 (0.840, 0.946) in Group C; 0.899 (0.805, 0.993), 0.972 (0.877, 1.000), and 0.964 (0.919, 1.000) in Group D]. The intra-reader agreement of radiologist B in evaluating the image noise, venous enhancement, image quality, and confidence of DVT was almost perfect [0.899 (0.787, 1.000), 0.972 (0.862, 1.000), and 0.964 (0.919, 1.000), respectively, in Group A; 0.884 (0.835, 0.933), 0.845 (0.767, 0.923), and 0.842 (0.715, 0.969), in Group B; 0.961 (0.916, 1.000), 0.893 (0.781, 1.000), and 0.899 (0.789, 1.000) in Group C; 0.969 (0.916, 1.000), 0.845 (0.733, 0.956), and 0.844 (0.680, 0.897) in Group D].

### Radiation dose

The CTDIvol and DLP for the non-enhanced group were 3.39 ± 0.58 mGy and 375.49 ± 68.78 mGy·cm, respectively. Likewise, the enhanced examination resulted in identical values for CTDIvol and DLP. The total CTDIvol and DLP were 6.40 ± 1.15 mGy and 750.98 ± 137.56 mGy·cm, respectively.

## Discussion

This study evaluated the indirect CTV of the lower extremity image quality of the AiCE, AIDR 3D, AiCE, and AIDR 3D algorithms combined with CE-boost(AiCE-boost, AIDR 3D-boost)and diagnostic confidence of four groups images in detecting DVT. The evaluation included an objective analysis of image indicators, including CT attention, image noise, SNR, and CNR. Additionally, subjective grading was applied to assess the image quality of lower extremities and diagnostic confidence. To our knowledge, this is the first research to combine the CE-boost technique and deep learning algorithm for CTV of the lower extremities. The study results revealed that CE-boost images demonstrated increased levels of venous vascular enhancement, SNR, and CNR compared to the enhanced images. Furthermore, CE-boost images have higher subjective scores and are more favorably accepted by radiologists than conventional enhanced images.

Sufficient venous enhancement played an essential role in facilitating the detection of DVT. In this research, the application of 80 kVp CTV with a reduced volume of contrast medium, in conjunction with either AIDR 3D or AiCE and the CE-boost method, resulted in a significantly increased level of venous enhancement compared to previous findings (158.0 ± 20.2 HU) [14]. The CT values of CE-boost images showed an improvement of approximately 1.3 times compared to the original images. Specifically, the degree of enhancement in the CE-boost images surpassed the CT values of the veins (range 152.7-175.0 HU) with ASIR-V at 70 kVp (2 mL/kg, CM = 320 mgI/mL) [2] and over CT values of the veins (range 146.4-154.4 HU) with filtered back projection (FBP) at 100 kVp (120 mL, CM = 370mgI/mL). The advantage of the CE-boost method is that it efficiently mitigates the issue of insufficient vascular enhancement, providing significant solutions for images with suboptimal venous enhancement. The mean enhancement of DVT was observed to be 51 HU or lower [19]. CE-boost can increase the difference in CT values between venous blood and thrombosis. Moreover, this holds essential clinical significance, particularly in individuals with small-diameter venous thrombosis, multiple venous thrombi, and compromised venous circulation.

The CE-boost technique shows promise in reducing the amount of contrast media and improving radiologists’ diagnostic confidence. Previous studies have demonstrated that the application of CE-boost significantly improves the detection of type II endoleaks following endovascular aortic aneurysm repair [7], shows efficacy in visualizing smaller diameter abdominal arteries and portal veins [9, 10] and peripheral pulmonary arteries [8]. In the present research, the CE-boost images exhibited improved visibility of the veins compared to the original images. Moreover, they accurately depicted the inferior vena cava and lower extremity vein thrombi. This technique can be iteratively applied to enhance the visibility of contrast for clinical diagnostic purposes without increasing the administration of iodine contrast and radiation dose. In addition, incomplete pixel matching between the unenhanced and enhanced images results in slight blurring of the vessel edges or tissue edges on the CE-boost images, which can be resolved by keeping the patient as stationary as possible during the unenhanced and enhanced scan.

This research revealed that AiCE algorithms were more effective at decreasing noise than AIDR 3D iterative algorithms, with AiCE providing the most significant advantage in image noise reduction during CE-boost. In theory, due to the incomplete match between the enhanced images and the non-enhanced image pixels, the CE-boost images will have greater noise than the original enhanced images. Our findings show that the noise of the AiCE-boost image was comparatively lower than that of AiCE; this discrepancy can be attributed to the implementation of a noise-reducing technique on the subtracted ionograms during the CE-boost process. Denoising of the iodine images of AIDR 3D is also performed during the CE-boost process. However, AIDR 3D-boost images had similarities to the noise observed in AIDR 3D. This can be attributed to the limited denoising effect of the CE-boost process on the iodine images, which cannot compensate for the excessive inherent system noise in the AIDR 3D images. The CE-boost images are influenced by the magnitude of the noise in the original images. In other words, the reconstruction algorithm influences image quality following CE-boost. The noise level of CE-boost images will be high if the noise of the original images is high. In previously published studies on abdominal CT angiography, CE-boost images using the forward projected model-based iterative reconstruction solution (FIRST) can obtain images with lower noise than FIRST [18], aligning with the findings of this research.

Opting for 80 kVp over 100 or 120 kVp in CT venography shifts the mean energy of the X-ray beam nearer to the K-edge of iodine (33.2 keV), thereby intensifying the photoelectric effect, augmenting vascular contrast in the iodine absorption spectrum, improving venous vessels visualization [18, 21]. Additionally, employing an iterative technique can effectively reduce excessive image noise caused by low kVp settings, enhancing both the subjective and objective quality of images and boosting radiologists’ confidence in diagnosing vein thrombosis.

Previous literature [2–4, 21] has demonstrated the advantages of improving vascular enhancement, particularly with lower tube voltages such as 100 kVp, 80 kVp, or 70 kVp when combined with iterative algorithms (MBIR, ASIR-V, etc.). In this study, AiCE and AiCE-boost images were superior to AIDR 3D and AIDR 3D-boost images in objective and subjective aspects. The reconstruction time of images using the AiCE algorithm can meet clinical needs. The average time to reconstruct a patient’s head images (0.5 mm for slice thickness and layer spacing) using AIDR 3D and AiCE was 27s and 44s, respectively [22]. In this study, the total time to reconstruct a patient’s CTV images (unenhanced + enhanced) was approximately (70–85 s) s for AIDR 3D, (145–160) s for AiCE.

In this research, the total DLP was 750.98 ± 137.56 mGy·cm, which was 58% lower than that of the conventional lower limb CT protocol with the application of dual-layer spectral detector CT at 120 kVp (1823.45 ± 512.68 mGy·cm) [23] and CTV on a 64-MDCT scanner at 120 kVp (1774.9 ± 426.0 mGy·cm) [24], and 38% lower than CTV using a 64-MDCT scanner at 100 kVp (1202 ± 273.5 mGy·cm) [24]. However, the total DLP was higher than comparable studies using 80 kVp (361.5 ± 66.2 mGy·cm) and higher than the study using 70 kVp (344.0 ± 45.4 mGy·cm) [2]. Considering CE-boost imaging requires non-enhanced images as well as enhanced images, the total DLP includes the exposure to non-contrast images. However, increasing the fixed SD of the ATCM or lowering the CT tube current combined with the AiCE algorithm can solve the problem of patients’ relatively high radiation dose. If unenhanced scan protocol was not to be used, the mean DLP of enhanced protocol (342.92 ± 20.05 mGy·cm) did not additionally increase the radiation dose to the patients. Furthermore, unenhanced images are necessary for patients with intravenous mass, mass involvement of veins, such as leiomyomas, fibroma of muscle, radiologist can determine whether the mass is enhanced and how much.

The current study also has several limitations. Firstly, this was based on a relatively limited sample of participants and was a retrospective single-arm study, which introduced potential biases in patient selection and data collection. To validate our findings and increase the generalizability of the results to other institutions and patient populations, a prospective study with a larger sample size in a multicenter setting is required. Secondly, the CE-boost technique is subjected to scanning protocol and requires the acquisition of both non-enhanced and enhanced images, which increases the patients’ radiation exposure. Therefore, it is recommended that the fixed standard deviation be increased to decrease radiation further. Thirdly, the study did not evaluate other reconstruction techniques, such as FBP, and model-based IR (e.g., FIRST). Due to the high noise level of FBP, it is no longer commonly used in clinical practice. FIRST images have higher quality and better detectability with low contrast, but the reconstruction time is too long, and the acceptance by radiologists is poor [25]. Despite its limitations, the study contributes to our understanding of the deep learning reconstruction algorithms and CE-boost technique in CT venography of lower extremities. However, further investigation is warranted for a more comprehensive understanding.

In conclusion, the study aimed to examine whether there is a significant difference in image quality between the HIR and DLR reconstruction algorithms on the conventional enhanced and CE-boost images of indirect CTV of lower extremities. The CE-boost technique can improve the overall image quality of lower extremities indirect CTV images and increase radiologists’ diagnostic confidence. Deep learning reconstruction algorithms with the CE-boost technique decreased the image noise and increased the CT values, SNR, CNR, and subjective image scores in low radiation dose CTV. DLR, and the integration of CE-boost (AiCE-boost) images yielded superior image quality compared to the other three datasets, making it more readily accepted by radiologists. DLR with CE-boost could be a valuable tool for improving the diagnosis of deep vein thrombosis and other venous conditions and potentially improve patient care and diagnostic accuracy in real-world clinical settings.

## Data Availability

The data of this study will be available by contacting the corresponding author.
